# The First Ant-Termite Syninclusion in Amber with CT-Scan Analysis of Taphonomy

**DOI:** 10.1371/journal.pone.0104410

**Published:** 2014-08-20

**Authors:** David Coty, Cédric Aria, Romain Garrouste, Patricia Wils, Frédéric Legendre, André Nel

**Affiliations:** 1 Muséum National d’Histoire Naturelle, Institut de Systématique, Evolution, Biodiversité, ISYEB, UMR 7205 CNRS UPMC EPHE, Paris, France; 2 Department of Natural History-Palaeobiology, Royal Ontario Museum, Toronto, Ontario, Canada; 3 Department of Ecology & Evolutionary Biology, University of Toronto, Toronto, Ontario, Canada; 4 CNRS UMS 2700, Muséum National d’Histoire Naturelle, Paris, France; University of Freiburg, Germany

## Abstract

We describe here a co-occurrence (i.e. a syninclusion) of ants and termites in a piece of Mexican amber (Totolapa deposit, Chiapas), whose importance is two-fold. First, this finding suggests at least a middle Miocene antiquity for the modern, though poorly documented, relationship between *Azteca* ants and *Nasutitermes* termites. Second, the presence of a *Neivamyrmex* army ant documents an in situ raiding behaviour of the same age and within the same community, confirmed by the fact that the army ant is holding one of the termite worker between its mandibles and by the presence of a termite with bitten abdomen. In addition, we present how CT-scan imaging can be an efficient tool to describe the topology of resin flows within amber pieces, and to point out the different states of preservation of the embedded insects. This can help achieving a better understanding of taphonomical processes, and tests ethological and ecological hypotheses in such complex syninclusions.

## Introduction

Ants and termites represent ecologically critical organisms in intertropical and subtropical ecosystems, impacting by their abundance, organization and variety of occupied niches the availability of nutrients as well as the composition of soils [1]–[5]. Although conspicuous and ecologically meaningful, the relationships between these key eusocial insects are sparsely documented. The data gathered so far have reported on ant predatory behaviour over termites [6]–[9] and/or termite nest (termitaria) occupation by ants [10]–[13]. The question of the antiquity of these relationships remains untackled, which overlaps with elucidating the age and stability of modern ‘hot spots’ of biodiversity occupied by these insects. Ants and termites are recorded since the early Cretaceous [14], [15], but there is yet no fossil record of interactions between these two taxa. This is despite the fact that ants are very common in the Neogene Neotropical and Eocene Baltic amber [16]. They can be found in syninclusions with numerous other insects of various groups, therefore giving possibilities to track the origin of extant behaviours involving these organisms in the fossil record [17], [18]. The term ‘syninclusion’ is therefore intended here in the sens of Koteja [19], for multiple organic inclusions in the same piece of amber, essential for understanding arthropods paleobehaviours in past environments.

**Figure 1:**
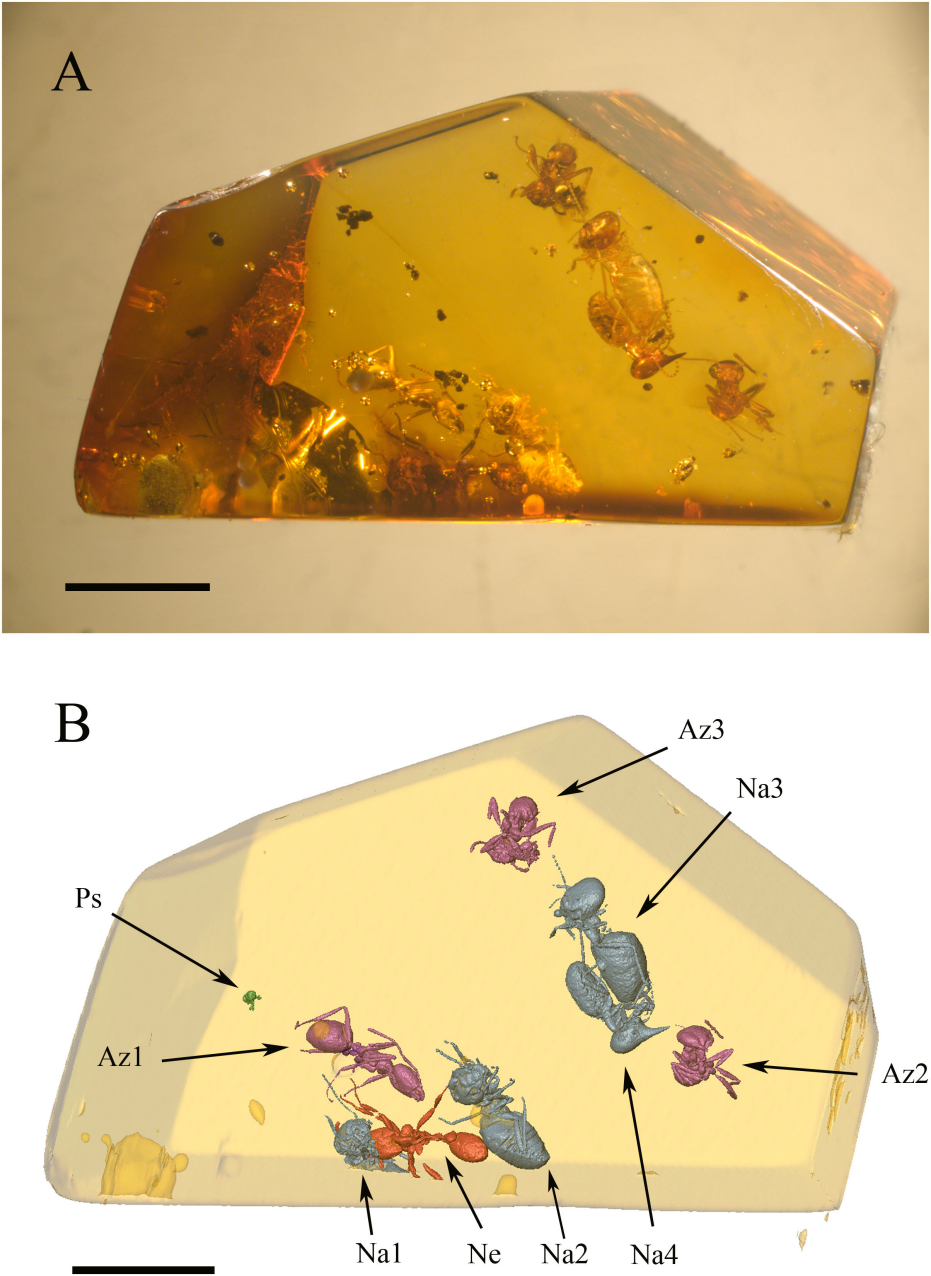
General configuration of the syninclusion. (A) Overview of the amber piece, under optical microscope. scale bar = 3 mm. (B) Three-dimensional replica of the same; colours define taxonomic groups, viz. purple for *Azteca* ants, blue for *Nasutitermes* termites, red for *Neivamyrmex* ant, green for small Psocodea. Labels: Az1, *Azteca* ant nearest to predation scene; Az2 and Az3, two other *Azteca* ants, both trapped in a flow distinct to that of the others inclusions and whose physical density matches that of the *Nasutitermes* soldier; Na1, *Nasutitermes* worker trapped between the *Neivamyrmex* mandibles; Na2, isolated *Nasutitermes* termite closest to predation scene; Na3, *Nasutitermes* worker with damaged gaster; Na4, *Nasutitermes* soldier; Ne, *Neivamyrmex* ant; Ps, Psocodea; Scale bar = 3 mm.

We aim here to describe the first syninclusion of termites and ants (for a review of syninclusions see [18]) in a piece of Mexican amber from the Totolapa deposit, together with an adult Psocodea (Figures 1A–B, S1, S2). The exceptional feature of this syninclusion lies in the fact that a raider ant (*Neivamyrmex*) and inquiline ants (*Azteca*) are entrapped together with *Nasutitermes* termites, thus ensuring that these genera were present at exactly the same time and shared at least a part of their ecological niche.

Using CT-scan analysis with the purpose to improve access to taphonomically concealed features, we discovered that our amber piece was the result of several different flows and that the preservation of internal organic structures differed between insects. If tomographic analyses have already been widely used for taxonomical studies of insects, reconstructions of their external and internal morphology [20]–[26], and to illustrate a syninclusion in amber [27], we also use here the CT-scan as a tool to analyze the results of taphonomical processes in an amber syninclusion.

## Material, Locality and Method

This amber piece was discovered in a batch of crude amber acquired by one of us (DC) from locals exploiting the Totolapa amber deposit (Salt River Mine). Later, the piece was offered to the Muséum National d’Histoire Naturelle de Paris (specimen MNHN.F.A49933). Totolapa is a village located in the central depression of Chiapas, 70 km south-east of Tuxtla Gutiérrez, the capital of Chiapas State. The Salt River amber mine, exploited since 2007 by Manuel Ramirez and his son Heriberto, is 1 km north of Totolapa, on the banks of the Salt River. The arthropod fauna collected by DC is currently under study. The age of the main Mexican amber locality, Simojovel, is still in debate, between Late Oligocene to Middle Miocene [28]–[33]. According to a geological map of the Instituto Nacional de Estatistica y Geografia [34] Totolapa amber would be Eocene in age, but a recent geological study of the Totolapa deposit suggests that the material originates from the Early Miocene Mazantic and Balumtum formations on top of Eocene marine facies [35]. As a matter of fact, Lambert et al. [36] suggested after a Carbon13 NMR spectroscopy study made on Baltic, Dominican and Mexican amber, that Simojovel and Totolapa amber came from the same palaeobotanical source; while Dominican amber, even if also closely related, shows more differences with both Mexican deposits. We therefore consider that the age of Totolapa amber is most probably between late Oligocene to middle Miocene and that the producing tree could also be *Hymenaea mexicana* [37] or a *Hymenaea* of undetermined species. Further geological studies are therefore needed to discover the precise age of Totolapa amber.

The original external surface of the amber piece has been removed by polishing; final lustration was done using diatomite powder. The specimens were examined under Nikon SZ10 and Olympus SZX9 stereomicroscopes. Photos were taken with an Olympus E-3 digital camera. Several digital pictures were reconstructed using Helicon Focus software.

X–ray tomography was realised in the AST-RX service (CT scan facility of the MNHN, UMS 2700), using a v|tome|x L240-180 from GE Sensing and Inspection Technologies phoenix|x-ray, with a X-Ray 180 KV/15 W nanofocus transmission tube, as well as a movable detector formed by a 20242 pixels (200 microns pixel). The voxel size of the reconstructed volume is 11.2 µm. 3D reconstructions and movies have been made using AVIZO 7.0 software. Variations in the material density in the amber piece are visible through changes of coloration from black (low density) to white (high density).

No permits were required for the described study.

### Systematic palaeontology

Identifications of the specimens were possible at the generic level but not at the specific level, for the reasons indicated below.

**Figure 2:**
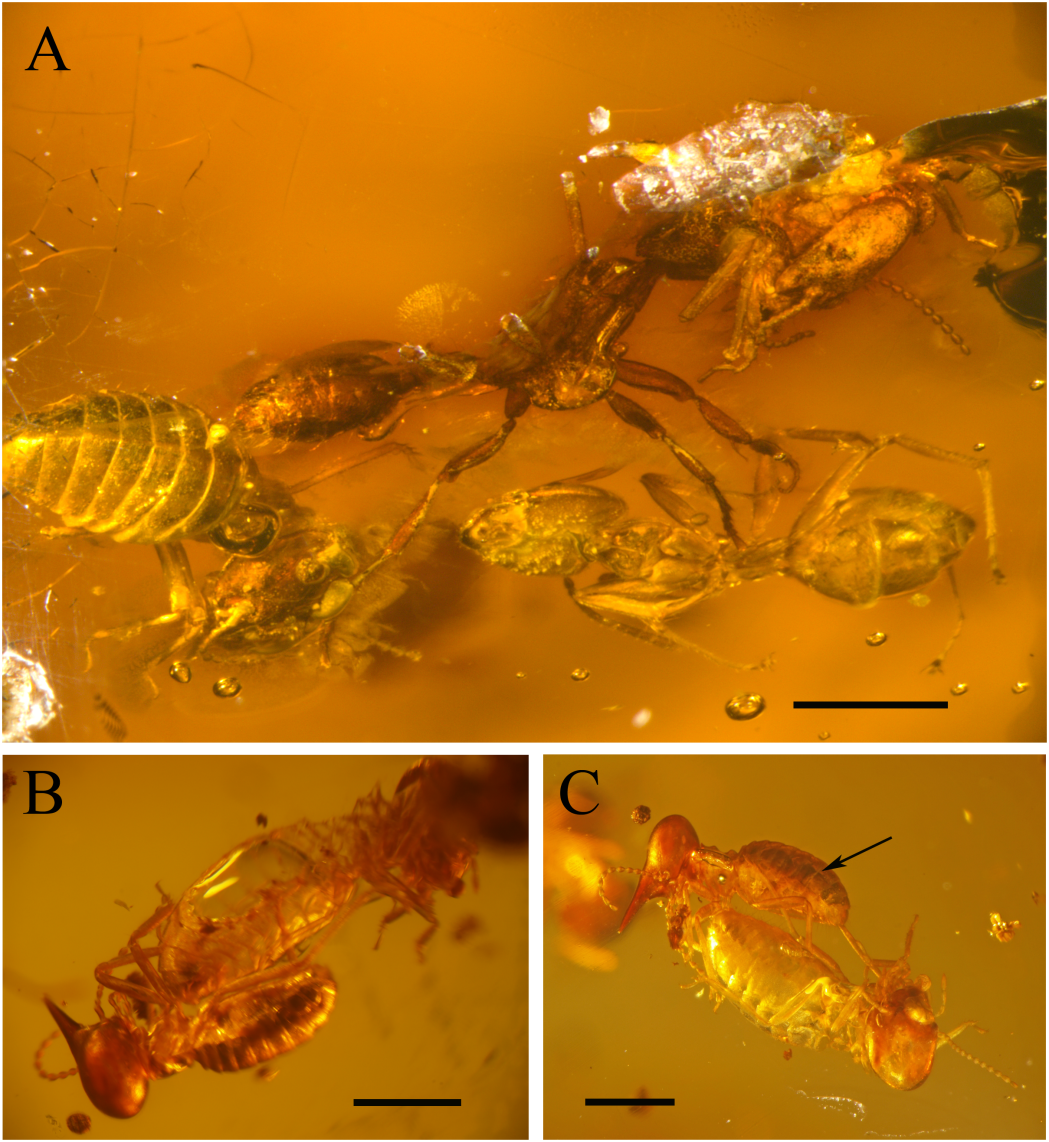
Details of the syninclusion. (A) General side view of the *Neivamyrmex* ant holding a *Nasutitermes* termite (Na1) between its mandibles, under optical microscope, scale bar = 1 mm. (B) detail of damaged gaster of *Nasutitermes* worker (Na3) closely contiguous to a *Nasutitermes* soldier (Na4), scale bar = 1 mm. (C) side view of closely contiguous *Nasutitermes* soldier (Na4) and worker (Na3), black arrow: digestive tube of *Nasutitermes* worker scale bar = 1 mm.

Order Isoptera Brullé, 1832; Family Termitidae Latreille, 1802; Subfamily Nasutitermitinae Hare, 1937; Genus *Nasutitermes* Dudley, 1890; *Nasutitermes* species (Figures 1B, 2A–C).

Comments. Typical workers and soldier termites assignable to *Nasutitermes* sp. by the following diagnostic characters: soldier with vestigial mandibles, with points; head capsule rounded, without constriction behind antennae; presence of a glabrous and narrow-tipped conical frontal tube (nasus); pronotum saddle-shaped and proctodeal segment not forming a loop on the right side of abdomen.

In Mexican amber, *Nasutitermes* was hitherto known only from imagos [38], to which our fossil soldier cannot be compared. From the two species known by soldiers in coeval Dominican amber, i.e. *N. electronasutus* Krishna, 1996 and *N.rotundicephalus* Krishna and Grimaldi, 1999, our fossil differs by its bare head as opposed to a head with long setae [39], [40]. Finally comparison of a new fossil *Nasutitermes* with the approximately 260 known modern species is highly difficult, given the absence of a reliable key and the uneven reliability of the various descriptions. We therefore do not ascribe our fossil to any particular species, and instead left it as *Nasutitermes* sp.

Order Hymenoptera Linné, 1758; Family Formicidae Latreille, 1809; Subfamily Dolichoderinae Forel, 1878; Genus *Azteca* Forel, 1878; *Azteca* species. (Figures 1A–B, 2A).

Comments. Dolichoderine ant with the following characters: nodiform petiole; unarmed hypostoma and propodeum; developed eyes; vertical first gastral tergite and anterior clypeal margin without a broad median concavity [41].

These *Azteca* ants will be described in a future paper, encompassing all the other *Azteca* present in the David Coty Totolapa amber collection.

Order Hymenoptera Linné, 1758; Family Formicidae Latreille, 1809; Subfamily Ecitoninae Forel, 1893; Genus *Neivamyrmex* Borgmeier, 1940; *Neivamyrmex* sp. (Figures 1A–B, 2A–C).

Comments. Ecitonine ant with the following diagnostic characters: eyes absent or reduced to an ommatidium; promesonotal suture absent or vestigial; antenna 12-segmented; antennal sockets fully exposed; absence of a preapical tooth on inner curvature of mid and hind pretarsal claws.

Only two fossil Ecitoninae, both from Dominican amber, are currently recorded: *Neivamyrmex ectopus* [42] and an undescribed army ant associated with a prey wasp pupa [43]. *Neivamyrmex ectopus* differs from our specimen in having a petiole with a subpetiolar process. Nevertheless, as the cuticle of our fossil specimen is badly preserved, and as we cannot reshape the amber piece (to preserve the syninclusion as a whole) in order to access further taxonomic details, we refrain from ascribing a new species.

### General description of the amber piece

Our amber piece is 1.6 cm long, 1.0 cm wide and 1.2 cm high. It contains three *Azteca* ants (specimens Az1, Az2, and Az3 in Figure 1B), one Neivamyrmex ant (specimen Ne in Figure 1B), four *Nasutitermes* termites (Na1, Na2, Na3, and Na4, in Figure 1B), and a Psocoptera (specimen Ps in Figure 1B). The *Neivamyrmex* ant (Ne) holds a minor termite worker (Na1) between its mandibles (Figure 2A). In their vicinity, we can find one of the *Nasutitermes* workers (Na2) an *Azteca* ant (Az1), and a Psocoptera (Ps). Further away are grouped together two contiguous *Nasutitermes* termites, a soldier (Na4) with preserved digestive tube (Figure 2C), and a worker (Na3) with the gaster partly damaged (Figure 2B). The two remaining *Azteca* ants (Az2 and Az3) stand aside from both groups, and one from the other. See Figure S1 for a 3D view of the syninclusion.

### CT-scan results

**Figure 3:**
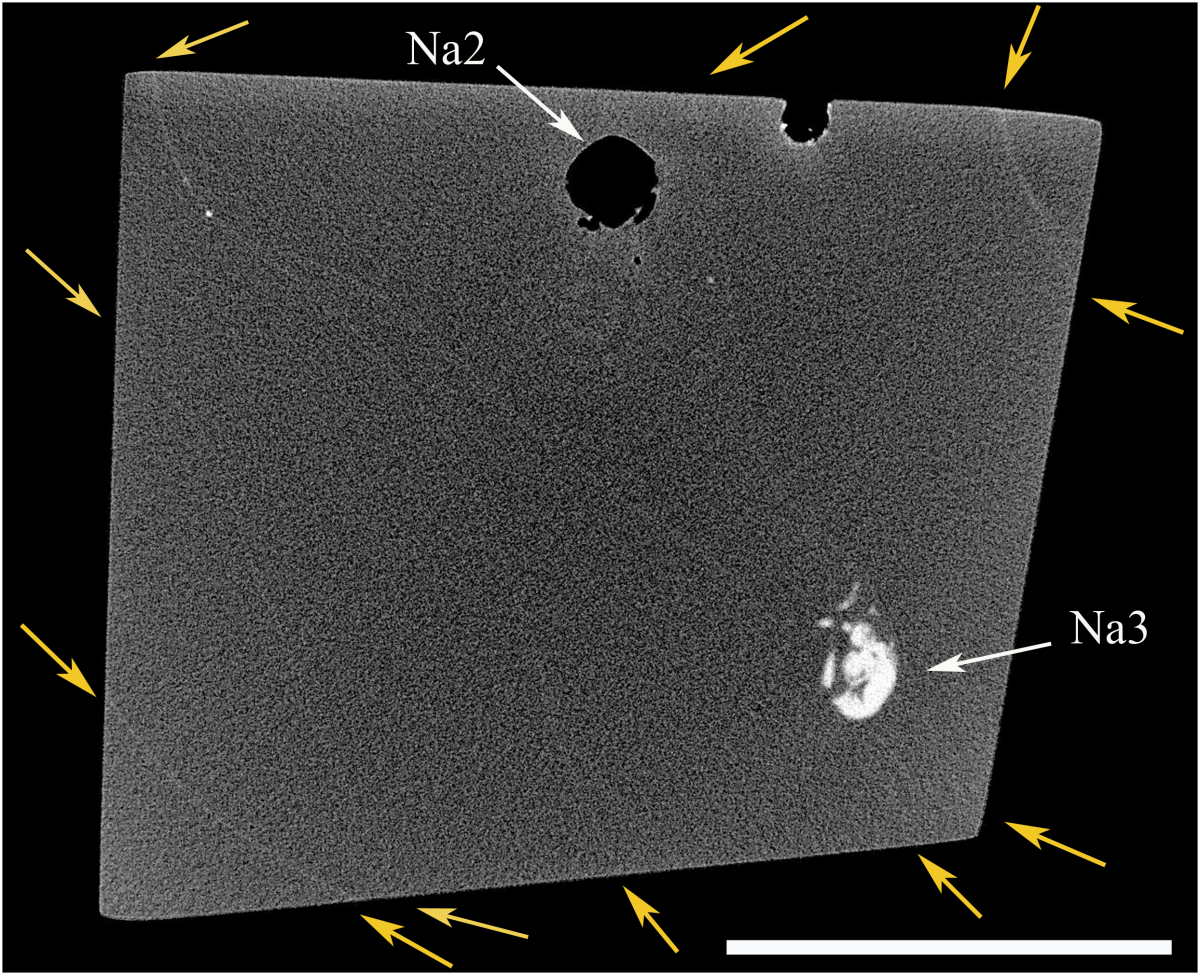
Virtual slicing from CT scan analysis showing flow boundaries. Yellow arrows shows starting and ending points of flows limits. Na2 and Na3 belong to the same flow, strong disparity in density matter between specimens from same taxonomic groups, scale bar = 5 mm.

X-ray tomographic analysis revealed that our amber piece is in fact made of eight distinct layers corresponding to different flows, and that the distribution of the insects does not reflect a synchronous event. The layers are delimited by sinuose surfaces whose intersections with the different tomographic slicing are rendered as sinuate lines (variations of matter density visible in the images, see Figures 3, and S2).

The fossil specimens are entrapped in two of the eight visible flows hereby identified (Figures 3, S2). Other flows are devoid of insect inclusions. The first flow with inclusions, herein named the ‘*predation* flow set’, contains the *Neivamyrmex* ant holding the minor termite between its mandibles (Ne+Na1), one *Azteca* ant (Az1), one *Nasutitermes* worker (Na2) the Psocoptera (Ps), and the two contiguous *Nasutitermes* termites (Na3+Na4). The second flow, named the ‘*Azteca* flow set’ contains the two isolated *Azteca* specimens (Az2 and Az3).

**Figure 4:**
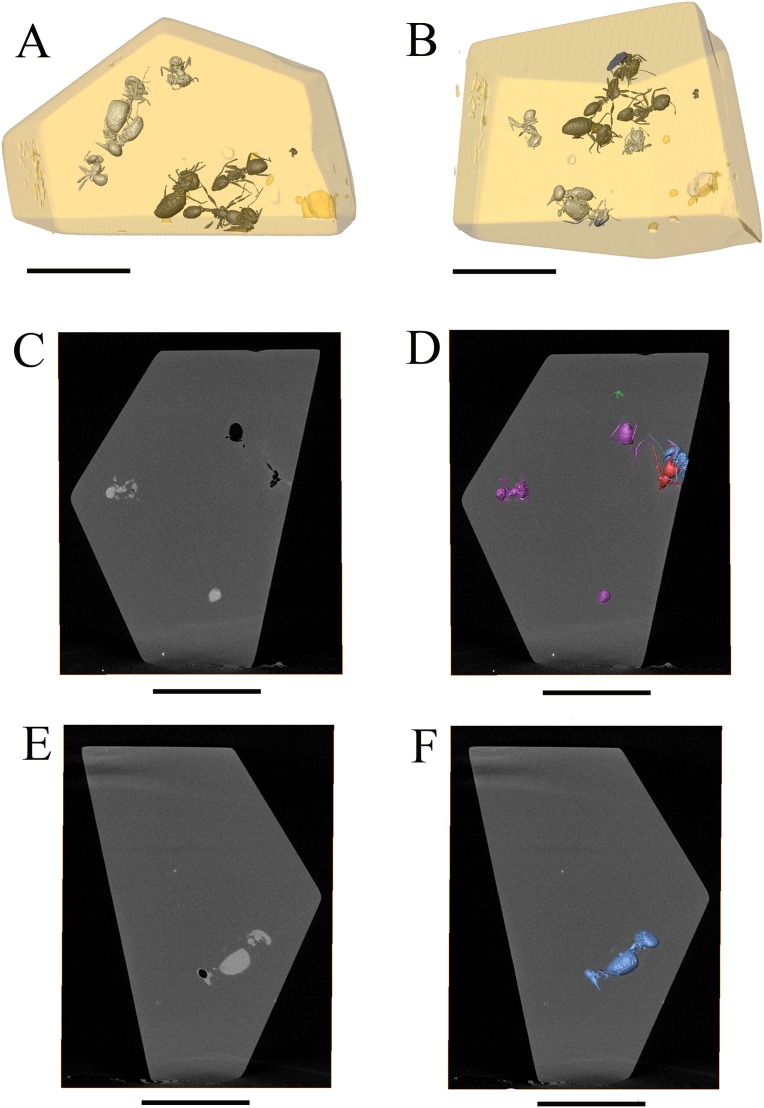
Density differences identified by CT scan. (A–B) Three-dimensional reconstruction artificially coloured representing two different density sets. ‘*Nasutitermes* soldier’ set in white, ‘*Neivamyrmex*’ set in black, as in CT-scan slices, Scale bar = 5 mm. (C) reslice of the original CT scan slice showing density differences between specimens. High density in white or light gray, low density in black or dark gray (darker than amber). Az2 and Az3 clearly denser than amber, Az1 and Ne empty structures, scale bar = 5 mm. (D) same slice as in B, but with 3D reconstruction of specimens, showing slice position in syninclusion, and specimen correspondences, scale bar = 5 mm. (E) reslice of original CT-scan slices, showing *Nasutitermes* soldier (Na4) and *Nasutitermes* worker (Na3), both denser than amber, scale bar = 5 mm. (F) same slice than E, but with 3D reconstruction of specimens, showing positions of specimens, scale bar = 5 mm.

CT-scan analysis also emphasised variations in the physical density of the specimens. Empty specimens appear in black (low density registered) on the slices obtained with the CT-scan. In our case, the *Neivamyrmex* ant (Ne), the minor termite worker trapped between its mandibles (Na1), one *Nasutitermes* worker (Na2), and one *Azteca* ant (Az1) appear as empty structures inside the amber piece (in black on the slices, see Figures 3, 4C). On the contrary, the *Nasutitermes* soldier (Na4), the *Nasutitermes* worker with the damaged gaster (Na3) and two *Azteca* ants (Az2 and Az3) appear as full structure, denser than the amber (in white and light gray on the slices, see Figures 3, 4C–F). Thus, two coherent density sets can be distinguished in our piece of amber: the ‘*Nasutitermes* soldier density set’ and the ‘*Neivamyrmex* density set’ (Figures 4A–B).

## Discussion

### Ants-termites interactions – palaeoecological interpretation of the syninclusion

In the Neotropics, *Nasutitermes* is often involved in relationships with ants, possibly in relation with the fact that it is the termite genus with the highest number of species building conspicuous nests [4], [11]. A total of 54 extant ant species have been reported living in the different stages of the *Nasutitermes* nests [11]. Among those, *Azteca* species (*A*. *chartifex* Forel, 1896 and *A*. *gnava* Forel, 1906) have been found living at the three categorized termitaria stages (active, decadent, abandoned). The implications of extant *Azteca* in the opportunistic occupation of termitaria have been briefly described elsewhere [10], [11], but the nature of interactions is mostly unknown.

In the modern Venezuelan forests, it seems that some ants (including undetermined *Azteca* spp.) occupy the nests of *Nasutitermes corniger* (Motschulsky, 1855) to protect themselves during the flooding events of the wet season [10]. During this temporary association, termites tolerate the predation of ants on their colony, as they themselves take the opportunity to feed on dead ants which constitute a valuable source of nitrogen. Nutrients flows occur both way between termites and ant in such association. Termites may also take benefits of the presence of inquilines ants in their nests to defend their common colony against predators [1], [44]–[46].

Cases of termites occupying parts of an active ant colony are also known [47]–[50]. Reasons of such cohabitations are generally unknown, although it has been noted that the contacts between ants and termites are rare in such cases, and qualified as neutral. Trager [50] mentioned that this type of association is frequent in the Neotropical region, involving different species of termites and ants.

Also called ‘The Huns of the insect world’ [51], all the modern species of army ants are major predators of both invertebrates and vertebrates [52]–[54]. They are also known to have a preference for preying on other eusocial insects, and ants in particular [49], [54]–[56]. Cases of predation of army ants on *Nasutitermes* are recorded in the Neotropics [57]. As mentioned by Brady [58], army ants ‘never hunt or forage solitarily’ but ‘dispatch a mass of cooperative, leaderless foragers to locate and overwhelm prey simultaneously’.

By phylogenetic inference, it is possible to say that our Mexican amber *Neivamyrmex* had the same behaviour and biology as its modern relatives (see [59], for inferences from recent fossil). The fact that this *Neivamyrmex* army ant holds a minor termite between its mandibles (Figure 2A) supports this hypothesis. Also, the gaster of the Na3 *Nasutitermes* termite is damaged, clearly showing traces of an ant bite (size and shape of the bite marks visible in Figure 2B). Lastly a phylogenetic inference shows that the *Azteca* ants were not predating the termites but more likely living with them in the same nest, as for their modern relatives. The presence of a *Nasutitermes* soldier (generally confined inside the nest and surging out of it for defence purpose), contiguous to the termite worker that exhibits an ant bite, also enhance the hypothesis (here again for an inference of a modern behaviour, see [59]) that the resin flowed close to a *Nasutitermes* nest.

This beam of evidences suggests that the fossil army ant present in our amber piece was part of a raid, during which the *Nasutitermes* termites and *Azteca* ants, sharing the same nest or interacting in some other degree might have been attacked, as it can typically occur in modern Neotropical settings.

The predation scene could also be the result of a peculiar type of scavenger behaviour, viz. when a predator is embedded while eating a dead insect only partly embedded in resin. However, the tomography shows that there is no discontinuity (a limit between two flows) between these two animals (see below for the study of flows using tomographic slices), which implies that they have been trapped in the same resin flow and thus invalidates this hypothesis.

The stress occurring during the embedment of the living ant together with a termite could have caused it to bite the termite randomly, but in this case, the presence of another termite partly eaten is not explained.

The presence of numerous *Azteca* ants (three specimens) and *Nasutitermes* termites (four specimens) in this small piece of amber, together with a Neivamyrmex ant reduces the possibility that *Azteca* ants and *Nasutitermes* termites have been randomly entrapped in the resin. It suggests that they were defending their common nest against an army ant ‘raid’ or interacting in some other degree while attacked. A ‘raid’ of army ants is always a strong perturbation for these eusocial communities, against which they have to defend, which bring many individuals out of their nest, therefore enhancing the probability to have many specimens of these different taxa entrapped together in a resin flow on the tree trunk.

### Interpretation of CT-scan results

#### Structure of the amber

**Figure 5:**
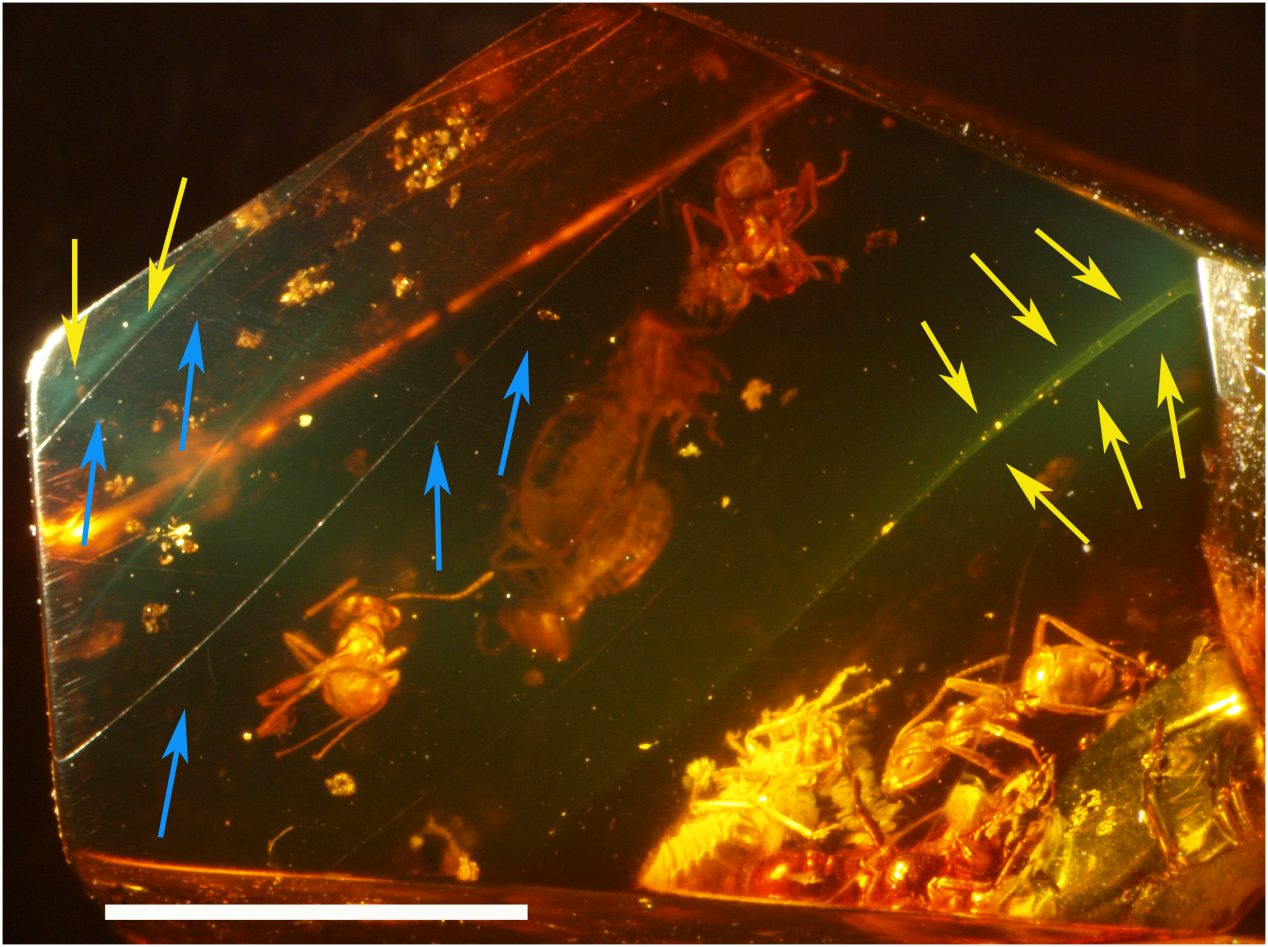
Flow margins visible under optic microscope. Yellow arrows: ‘green veils’ representing limits between two flows, blue arrows: ‘sinuate line’ visible on surface of the amber piece, scale bar = 5 mm.

The fact that amber pieces are almost always the result of several flowing event has been pointed out by various authors [60], [61]. Although the flow boundaries are partly visible at the surface of the amber piece (Figure 5), they are within the piece only detectable through greenish or brownish translucent surfaces, which appear under some particular view points, and light orientations (Figure 5). The CT-scan analysis therefore represents an informative enhancement allowing the clear mapping of the topology of each flow (Figure S2).

We discard the possibility that those sinuate lines could be cracks that may have occurred during the biostratinomy or the diagenesis of the amber, for two main reasons: 1) cracks never form smooth sinuate surfaces running across all the amber pieces; 2) they never follow the shape of inclusions but often damage them.

The surfaces of the flows appearing in CT-scan slices are always denser (i.e. lighter in the CT scan images) than the surrounding amber (also observed on other CT-scan slices of different amber pieces), suggesting that micro-particles, denser than the resin (not visible under optic microscope), deposited onto the amber surface before the arrival of the succeeding flow, or that a very thin layer of amber is harder at the surface contact between the two flows, as the resin was consolidated by polymerisation through sunlight and wind. The flow surfaces are nonetheless very weakly defined on the CT-scan slices and mostly not visible under optic microscope, possibly suggesting that the delays between flows might have been extremely short to limit the effects of polymerization on the flow surface, and avoid the deposition of dusts and debris on the fresh resin. It would therefore be likely that at least some relative degree of relation could exist between the density level of flow margins (in CT-scan imaging) and the time they were exposed to external elements, but further study on other material will be necessary to confirm such a hypothesis.

#### Differences of density between specimens

Since the relative density of the insects is unrelated to their taxonomy (*Azteca* ants and *Nasutitermes* termites are present in different density sets), and since there is a clear spatial homogeneity between the two density groups (Figures 4A–B), variations of physical density between the specimens themselves is likely to express taphonomical disparities. This may be related to differences in the preservation of the cuticle and the inner organic structures [61], and differences between the insects before their entombment in the fresh resin, i.e. dead versus living animals, animals with or without filled digestive tubes, etc. As a matter of fact, insects in amber are frequently empty, since most of the internal organic content is anaerobically degraded, as a result of autolysis and the activity of endogenous bacteria [62], [63]. On the other hand, the preservation of internal structures (i.e. the digestive tube in the gaster of the *Nasutitermes* soldier, Figure 2C) may be due to a phenomenon of dehydration before the complete embedment in resin, as it has been shown that a pre-entombment dehydration of the insects inhibits the latter degradation process of their body inside the resin [64], [65].

Following this logic, it would therefore be possible that the specimens of the ‘*Nasutitermes* density set’ where embedded already dead, while the specimens of the ‘*Neivamyrmex* density set’ where embedded alive.

It has to be noted that the presence of a white aureole surrounding the body of inclusions, considered to be a foam of microscopic bubbles by Mierzejewski [66] and Weitschat and Wichard [67] can also help in some cases to reveal which insects where trapped alive or dead in the resin [61], as it is possibly the result of an early diagenesis reaction between fluids, produced by decay and decomposition of labile tissues, with sugar and terpenes in the resin. However, such foams are rare in Mexican and Dominican ambers. In the present case, no foam is visible around our specimens to help us in our analysis.

The two homogenous density groups are not distinguishable under optic microscope. Some preserved internal organs are visible, but the level of preservation of the cuticles is difficult to evaluate. One particularly odd case is the *Nasutitermes* with the damaged gaster (Na3) as this gaster is obviously empty of any kind of internal structures, while CT-scan images show that its whole body is denser than the amber. The fact that the damaged part of the gaster exhibits a bite mark caused by an ant, and is entirely covered by what seems to be the edge of an air bubble (Figure 2B) strongly suggests that this specimen was dead before entombment in fresh resin. In fact, regarding what we mentioned above, if the two closely contiguous *Nasutitermes* (Na3 and Na4) are likely to have been both dead before entombment in the resin, the similarity of their density levels may not have the same taphonomical origin, and remain to be elucidated for specimen Na3 (*Nasutitermes*).

Biostratinomic processes (the period between the moment when resin is exuded from the tree to the moment when it is buried in sediment) could also be responsible for these differences in matter density, as great differences can occur between flows in term of duration of the flowing event (viscosity), degree of humidity of the air, level and time of exposure to air and to UV, etc. Such variations can therefore create disparities in the taphonomic process between inclusions present in different flows.

If we therefore cross-compare the density distribution between specimens with the different flows in which they were embedded, our amber piece shows that the two density groups almost fit with the distribution of the specimens inside the flows, except for the closely contiguous *Nasutitermes* soldier and the *Nasutitermes* worker with a damaged gaster, as they share the same density level as the two *Azteca* ants that belong to a different flow. Regarding what has been mentioned above, this result can be due to differences between the insects themselves before entombment in the resin.

## Conclusion

Our study provides evidence that some degree of relationship between *Azteca* ants and *Nasutitermes* termites might have already existed in Central America during the late Oligocene-middle Miocene period, together with the predation of army ants on other eusocial insects in the same community. However, the condition that led to the apparition of such interactions and their stability through time are still to be elucidated.

We also show here that beside anatomic reconstruction, CT-scan imaging can be used to study the taphonomy of syninclusions by allowing a more exhaustive description of resin flow topology and sequences, as well as a ‘cartography’ of density patterns of biotic inclusions. The main question to further address is to know in which measure both flow structures of the amber pieces and physical density variations of insect bodies can help to further reconstruct necrolysis, biostratinomic and diagenetic processes that occurred in the amber and its inclusions.

## Supporting Information

Figure S1 **PDF 3D of the syninclusion.**
(PDF)

Figure S2 **Movie of the syninclusion, showing the different flows of the resin that comprise the amber piece.**
(MPG)
