# Supplementary material for: The First Ant-Termite Syninclusion in Amber with CT-Scan Analysis of Taphonomy
Source: PLoS One. 2014 Aug 20;9(8):e104410. doi: 10.1371/journal.pone.0104410 (PMC4139309; doi:10.1371/journal.pone.0104410)
Supplement: Figure S1 — PDF 3D of the syninclusion. (PDF) [file pone.0104410.s001.pdf]

ACCÈS SCIENTIFIQUE À LA TOMOGRAPHIE À RAYONS X

Specimen : A Mexican amber syninclusion : Azteca ants and Nasutitermes termites together with a Neivamyrmex army ant.

Resolution : 11.2 μm
